# Nitric oxide generated by nitrate reductase increases nitrogen uptake capacity by inducing lateral root formation and inorganic nitrogen uptake under partial nitrate nutrition in rice

**DOI:** 10.1093/jxb/erv030

**Published:** 2015-03-17

**Authors:** Huwei Sun, Jiao Li, Wenjing Song, Jinyuan Tao, Shuangjie Huang, Si Chen, Mengmeng Hou, Guohua Xu, Yali Zhang

**Affiliations:** ^1^State Key Laboratory of Crop Genetics and Germplasm Enhancement, Key Laboratory of Plant Nutrition and Fertilization in Low-Middle Reaches of the Yangtze River, Ministry of Agriculture, Nanjing Agricultural University, Nanjing 210095, China; ^2^Tobacco Research Institute, Chinese Academy of Agricultural Sciences (CAAS), Qingdao 266101, China

## Abstract

Increasing evidence shows that partial nitrate nutrition (PNN) can be attributed to improved plant growth and nitrogen-use efficiency (NUE) in rice. Nitric oxide (NO) is a signalling molecule involved in many physiological processes during plant development and nitrogen (N) assimilation. It remains unclear whether molecular NO improves NUE through PNN. Two rice cultivars (cvs Nanguang and Elio), with high and low NUE, respectively, were used in the analysis of NO production, nitrate reductase (NR) activity, lateral root (LR) density, and ^15^N uptake under PNN, with or without NO production donor and inhibitors. PNN increased NO accumulation in cv. Nanguang possibly through the *NIA2*-dependent NR pathway. PNN-mediated NO increases contributed to LR initiation, ^15^NH_4_^+^/^15^NO_3_^–^ influx into the root, and levels of ammonium and nitrate transporters in cv. Nanguang but not cv. Elio. Further results revealed marked and specific induction of LR initiation and ^15^NH_4_^+^/^15^NO_3_^–^ influx into the roots of plants supplied with NH_4_^+^+sodium nitroprusside (SNP) relative to those supplied with NH_4_^+^ alone, and considerable inhibition upon the application of cPTIO or tungstate (NR inhibitor) in addition to PNN, which is in agreement with the change in NO fluorescence in the two rice cultivars. The findings suggest that NO generated by the NR pathway plays a pivotal role in improving the N acquisition capacity by increasing LR initiation and the inorganic N uptake rate, which may represent a strategy for rice plants to adapt to a fluctuating nitrate supply and increase NUE.

## Highlight

NO generated by nitrate reductase plays a pivotal role in improving N-use efficiency by increasing lateral root initiation and inorganic N uptake, representing a strategy for rice to adapt to fluctuating nitrate supply.

## Introduction

Nitrogen (N) nutrition affects all levels of plant functions, including metabolism resource allocation, growth, and development (Crawford, 1995; Stitt, 1999). Plants have the potential to adapt to dramatic fluctuations in N availability by modulating their nutrient acquisition capacity and by altering their metabolism and morphology, such as their root architecture (Crawford, 1995; López-Bucio *et al.*, 2003; Song *et al.*, 2013*a*; Manoli *et al.*, 2014). A dual effect of external nitrate on root system development has been demonstrated in the model plant *Arabidopsis thaliana*: (i) localized stimulation of lateral root (LR) elongation at the site of contact with a nitrate-rich supply and (ii) systemic inhibition of lateral primordia by uniformly high nitrate concentrations during the post-emergence stage (Zhang and Forde, 1998; Zhang *et al.*, 1999; Linkohr *et al.*, 2002; Zhang *et al.*, 2007; Ruffel *et al.*, 2011; Forde, 2014). Several known pathways involve microRNAs, transcription factors, hormonal signals, and nitrate transporters with a dual affinity for nitrate and auxin (Remans *et al.*, 2006; Chiou, 2007; Gifford *et al.*, 2008; Krouk *et al.*, 2010; Vidal *et al.*, 2010; Gojon *et al.*, 2011; Ruffel *et al.*, 2011; Trevisan *et al*., 2011, 2012a, *b*; Xu *et al.*, 2011; Zhao *et al*., 2011, 2012; Nischal *et al.*, 2012; Zhao *et al.*, 2013; Alvarez *et al.*, 2014; Begheldo *et al.*, 2014; Manoli *et al.*, 2014; Zeng *et al.*, 2014). However, our understanding of how plants sense external nitrate conditions and the signal transduction system that influences root system development and nutrient acquisition capacity remains limited.

Nitric oxide (NO) is a signalling molecule involved in many physiological processes during plant development and nutrient assimilation. Several lines of evidence suggest that NO is involved in the growth and development of the root system (Pagnussat *et al.*, 2003; Correa-Aragunde *et al.*, 2004,^2006^; Lombardo *et al.*, 2006; Stöhr and Stremlau, 2006; Guo *et al.*, 2008; Chen *et al.*, 2010; Wang *et al.*, 2010; Fernández-Marcos *et al.*, 2011; Meng *et al.*, 2012; Niu *et al.*, 2013; Wang *et al.*, 2013). Correa-Aragunde *et al.* (2004) suggested that auxin and NO are involved in a linear signalling pathway during the LR formation process in tomato. Several recent reports highlighted a role for NO in N assimilation and suggested that it is an important regulator of the first steps in the nitrate-sensing pathway (Du *et al.*, 2008; Jin *et al.*, 2009; Rosales *et al.*, 2010; Sanz-Luque *et al.*, 2013; Frungillo *et al.*, 2014). For example, nitrate reductase (NR) in the leaves of wheat was inhibited by NO (Rosales *et al.*, 2010), but was activated in cabbage (Du *et al.*, 2008). Indeed, the inhibition or activation of NR by NO in tomato roots may depend on the nitrate concentration (Jin *et al.*, 2009). However, the mechanisms of NO-regulated N uptake in response to nitrate fluctuation remain poorly understood.

NO synthase (NOS) and NR are two potential enzymatic sources of NO production in plants. Plant NOS has not yet been identified (Crawford *et al*., 2006; Moreau *et al*., 2008, 2010; Gas *et al.*, 2009; Gupta *et al.*, 2011), although experiments using inhibitors of the animal NOS enzyme have provided some evidence of the role of the L-arginine pathway in NO production (Zhao *et al.*, 2007). NR is one of the most important sources of NO in plants, and the nitrate concentration in the rooting medium can affect the amount of NO via mediation of NR activity (Yamasaki *et al.*, 1999; Meyer *et al.*, 2005; Yamasaki, 2005). Studies on *Arabidopsis* and *Vicia faba* confirmed that the relative and absolute levels of nitrate and nitrite were key determinants of NR-induced production of NO (Vanin *et al.*, 2004). Moreover, the localization of mRNA for NR in the root apex corresponded to the major sites of NO accumulation, as seen in *Arabidopsis* (Stöhr and Stremlau, 2006), suggesting that these NR genes represent pivotal elements of a finely tuned NO homeostasis and signalling system. NO is a nitrate-related signal generated by the NR pathway to regulate root system architecture (Zhao *et al.*, 2007; Manoli *et al.*, 2014; Trevisan *et al.*, 2014). However, the role of NO in the nitrate signalling pathway requires further investigation.

Rice (*Oryza sativa* L.), one of the most important staple food crops globally, is traditionally cultivated under flood conditions. Although ammonium (NH_4_^+^) is preferred over nitrate as the form of N as a nutrient in rice, rice roots are exposed to partial nitrate nutrition (PNN) due to nitrification in the rhizosphere (Li *et al.*, 2008). There is increasing evidence that PNN improves rice growth by inducing N uptake and assimilation, as well as the formation of adventitious roots and LRs (Qian *et al.*, 2004; Duan *et al*., 2007*a*, *b*; Cao *et al.*, 2008; Li *et al.*, 2008; Zhang *et al.*, 2011; Song *et al.*, 2011a, *b*, 2013*a*, *b*). In previous studies, seven rice cultivars with high N-use efficiency (NUE) and three with low NUE were selected from 177 japonica rice cultivars to study their responsiveness to PNN (Duan *et al.*, 2007*a*; Zhang *et al.*, 2008; Song *et al.*, 2011*a*). Under hydroponic conditions, five of the seven rice cultivars with high NUE were sensitive to PNN (Duan *et al.*, 2007*b*), and three rice cultivars with low NUE were insensitive to PNN (Song *et al.*, 2011*a*). Moreover, experiments using rice cultivars with contrasting NUE demonstrated that the increase in NUE caused by PNN is attributable to improved N uptake (Duan *et al.*, 2007*b*), suggestive of a possible relationship between PNN and NUE. It was hypothesized that NO plays a key role in the improved NUE of rice as an evolutionary adaptation to nitrate nutrition based on the following observations: (i) significant induction by PNN of nitrate concentrations in rice cultivars with high rather than low NUE relative to treatment with NH_4_^+^ alone (Song *et al.*, 2011*a*, b); and (ii) significant induction by PNN of auxin accumulation, and formation of adventitious roots and LRs in rice cultivars with high NUE (Song *et al.*, 2011b, 2013a). Auxin showed common stages with NO in the signal transduction cascade, which resulted in auxin-induced adventitious root and LR formation (Pagnussat *et al.*, 2003; Correa-Aragunde *et al.*, 2004). In this study, the role of NO in the regulation of rice LR formation and N-uptake capacity in response to PNN was examined in two rice cultivars with contrasting nitrate responses and N-uptake efficiencies. It was proposed that a high NUE in rice plants was attributable to NO generated by NR, which induces LR formation and inorganic N uptake.

## Materials and methods

### Plant materials and growth

Two rice cultivars (cvs Nanguang and Elio) were selected from 177 japonica rice cultivars based on their similar growth patterns and differential responses to N application in field trials conducted in 2003 and 2004 (Zhang *et al.*, 2007, 2008). Cv. Nanguang was identified as a high-nitrate-response and high-NUE cultivar, while cv. Elio was a low-nitrate-response and low-NUE cultivar (Duan *et al*., 2007*a*, b; Zhang *et al.*, 2009).

Cvs Nanguang and Elio were grown in a greenhouse under natural light at day/night temperatures of 30 °C/18 °C. Seven-day-old seedlings of uniform size and vigour were transplanted into holes in a lid placed over the top of pots (four holes per lid and three seedlings per hole). Nutrient solutions varying from a quarter to half-strength were applied for 2 d, after which full-strength nutrient solution was applied for an additional 14 d. Seedlings were subjected to two NH_4_^+^-N/NO_3_^–^-N ratios, namely 100/0 (NH_4_^+^) and 75/25 (PNN), by adding 1.43mM in the form of (NH_4_)_2_SO_4_ or a mixture of (NH_4_)_2_SO_4_ and NH_4_NO_3_. The chemical composition of the International Rice Research Institute (IRRI) nutrient solution was (mM): 0.3 KH_2_PO_4_, 0.35 K_2_SO_4_, 1.0 CaCl_2_, 1.0 MgSO_4_·7H_2_O, 0.5 Na_2_SiO_3_, and (μM) 20.0 Fe-EDTA, 9.0 MnCl_2_, 0.39 (NH_4_)_6_Mo_7_O_24_, 20.0 H_3_BO_3_, 0.77 ZnSO_4_, and 0.32 CuSO_4_; pH 5.5. A nitrification inhibitor (dicyandiamide, 7.0 μM) was added to each pot to prevent NH_4_^+^ oxidation. The nutrient solution was replaced daily with fresh solution. Nitrate was not detected in medium containing NH_4_^+^ alone. Rice plants were harvested 14 d after treatments. Each treatment consisted of four replicates arranged in a completely randomized design to avoid edge effects. In addition, all experiments included three independent biological replicates.

Preliminary experiments were conducted to determine the final amount of pharmacological application for cvs Nanguang and Elio. Pharmacological responsiveness to sodium nitroprusside (SNP) and tungstate (Tu) differed between the two rice cultivars (Supplementary Figs S1, S2 available at *JXB* online). For example, application of 1 μM or 2.5 μM SNP in addition to sole NH_4_^+^ nutrition induced an LR density of cv. Nanguang to a similar level to PNN treatment. However, the application of SNP (≥5 μM) induced LR density in cv. Elio comparable with that of sole NH_4_^+^ nutrition. Similarly, application of 50 μM Tu in addition to PNN decreased the LR density of cv. Nanguang to a similar level to sole NH_4_^+^ treatment. However, application of 100 μM Tu decreased the LR density of cv. Elio compared with PNN. Thus, different concentrations of SNP (2.5 μM for cv. Nanguang and 5 μM for cv. Eilo) and Tu (50 μM for cv. Nanguang and 100 μM for cv. Eilo) were applied in subsequent experiments. In addition, 80 μM 2-(4-carboxyphenyl)-4,4,5,5-tetramethylimidazoline-1-oxyl-3-oxide (cPTIO) was applied to the plant growth media.

### Measurement of LR density and LR primordia number

As reported previously, PNN increased the formation of adventitious roots and LRs in cv. Nanguang (Song *et al.*, 2011*b*). During shorter durations of the experiments (14 d), LR formation increased significantly but adventitious root formation was unaffected (Song *et al.*, 2013*a*). Thus, LR density and primordia were selected for subsequent, detailed analyses.

In this study, stages of LR development followed Malamy and Benfey (1997), with stages I–XII grouped here as unemerged primordia. The primordia of LRs were classified into unemerged and emerged primordia. An emerged LR primordium longer than 0.5mm (visible to the naked eye) was considered an LR, and was referred to as being activated (Song *et al.*, 2013*a*). To visualize the development of LRs, *pDR5::GUS* transgenic rice plants were exploited. After the roots were stained in GUS (β-glucuronidase) buffer solution, it was simple to count the number of primordia and LRs. The scaleplate on the stereomicroscope (Olympus Optical Co. Ltd, Tokyo, Japan) simplifies determination of the length of emerged primordia and LRs. Seminal root length was measured with a ruler, and LR density was calculated as LR number divided by seminal root length.

### Measurements of NO in the roots

NO was assayed using DAF-FM DA (diaminofluorescein-FM diacetate) and epifluorescence microscopy. Roots were loaded with 10 μM DAF-FM DA in 20mM HEPES-NaOH buffer (pH 7.5). After incubating in darkness for 30min, the roots were washed three times with fresh buffer and immediately visualized (OLYMPUS MVX10 stereomicroscope, with a colour CCD camera, excitation at 488nm, emission at 495–575nm). Signal intensities of green fluorescence in the images were quantified according to the method of Jin *et al.* (2011) using Photoshop software (Adobe Systems). Data are presented as mean fluorescence intensities.

### Determination of total N concentration and nitrate reductase activity (NRA)

The total N concentration in plants was determined using the Kjeldahl method (Li *et al.*, 2008). Maximum and active NR activities (NRAmax and NRAact) were measured in fresh roots using the method described by Li *et al.* (2008).

### Determination of ^15^N uptake rate

The ^15^N influx rate was assayed as described previously (Orsel *et al.*, 2006) for hydroponically grown plants. Seedlings were grown in the presence of 1.43mM NH_4_^+^ for 14 d and then N starved for 3 d. The plants were transferred to 0.1mM CaSO_4_ for 1min, then to a complete nutrient solution with the same N concentration (1.43mM) containing ^15^NH_4_^+^, ^15^NH_4_^+^/^15^NO_3_^–^ (PNN),^ 15^NH_4_^+^+SNP (2.5 μM, cv. Nanguang; 5 μM, cv. Eilo), and ^15^NH_4_^+^/^15^NO_3_^–^ (PNN)+cPTIO (80 μM for two cultivars) for 10min, and finally to 0.1mM CaSO_4_ for 1min. The ^15^N abundance in each fraction was determined using a MAT25 isotope mass spectrometer. NH_4_^+^ was labelled by [atom% ^15^N: (^15^NH_4_)_2_SO_4_, 40%] and PNN was labelled by [atom% ^15^N: (^15^NH_4_)_2_SO_4_, 40%/^15^NH_4_^15^NO_3_, 40% (50/50)] during the treatments.

### Quantitative reverse transcription–polymerase chain reaction (qRT–PCR)

Total RNA was isolated from the roots of rice seedlings. RNA extraction, reverse transcription, and qRT–PCR were performed as described previously (Tang *et al.*, 2012). Amplification of real-time quantitative PCR products was performed with a single-colour Real-Time PCR Detection System (MyiQ Optical Module; Bio-Rad) in a reaction mixture of 20 μl of SYBR Green master mix (SYBR Premix Ex Tag TMII; TaKaRa Bio; http://www.takara-bio.com) according to the manufacturer’s instructions (TaKaRa Biotechnology). The choice of the reference gene (*OsACT*) was supported by Tang *et al.* (2012). Primers and gene locus numbers for the *NIA1*, *NIA2*, *NOA*, *AMT1*, *AMT2*, *AMT3*, *NRT2*, and *NAR2* genes are listed in Supplementary Tables S1 and S2 at *JXB* online.

### Data analysis

Experimental data were pooled to calculate means and standard errors (SE), and were analysed by one-way analysis of variance (ANOVA) followed by least significant difference (LSD) to determine the significance of differences between individual treatments. All statistical procedures were conducted using SPSS ver. 11.0 (SPSS Inc., Chicago, IL, USA). In all analyses, *P*<0.05 was considered to indicate statistical significance.

## Results

### LR density and NO increased in cv. Nanguang in response to PNN relative to sole NH_4_^+^ nutrition

**Fig. 1.**
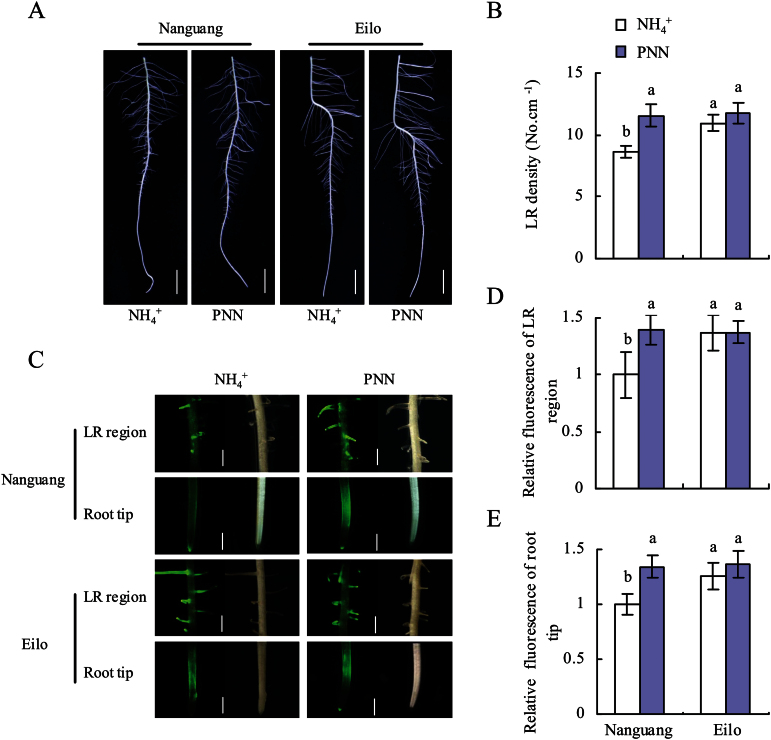
Lateral root (LR) development and NO accumulation in the tip and LR regions of seminal roots in cvs Nanguang and Elio. (A) LR morphology in the seminal root; scale bar=1cm. (B) LR density in the seminal root. (C) NO production, as indicated by green fluorescence, in representative roots. Scale bar=1mm. (D and E) NO production expressed as the fluorescence intensity relative to the same root region of Nanguang under NH_4_^+^ supply. Seedlings were grown under NH_4_^+^ and partial nitrate nutrition (PNN) for 14 d in hydroponic media. Data are means ± standard error (SE), and bars with different letters indicate significant differences at *P*<0.05, as determined by ANOVA.

As reported previously (Song *et al.*, 2011a, *b*, 2013*a*), LR density of the seminal root in the high-nitrate-response cultivar Nanguang, but not the low-nitrate-response cultivar Elio, increased significantly under PNN conditions compared with NH_4_^+^ alone (Fig. 1A, B). The NO-associated green fluorescence in both the root tip and LR regions of cv. Nanguang increased under PNN treatment, but no difference was observed in cv. Elio (Fig. 1C). Quantification of the fluorescence signal intensities (Fig. 1D, E) indicated an ~34% increase in the NO content in the LR region and the root tip of cv. Nanguang under PNN conditions compared with NH_4_^+^ treatment. These results indicated that NO production in the root tip and LR regions of cv. Nanguang under PNN conditions was higher than that with NH_4_^+^ treatment.

### NO participated in PNN-induced LR formation in cv. Nanguang but not in cv. Eilo

**Fig. 2.**
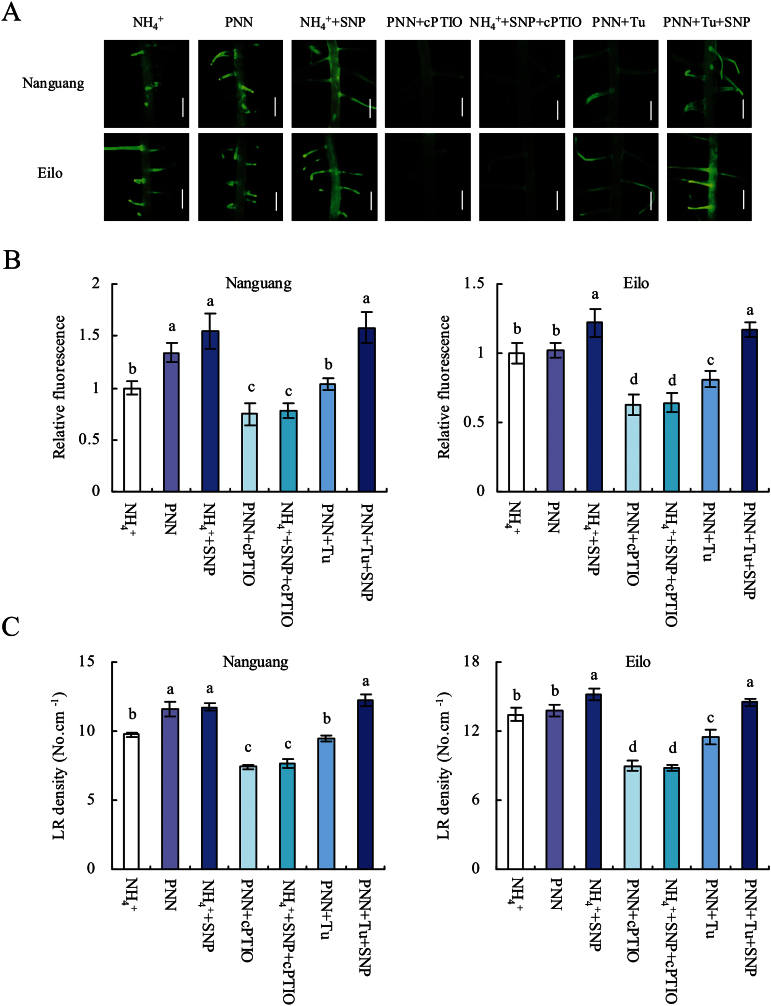
Lateral root (LR) development and NO accumulation in the LR region of seminal roots in cvs Nanguang and Elio. (A) NO production in the LR region is shown as green fluorescence. (B) NO production expressed as the fluorescence intensity relative to the LR region of cvs Nanguang and Elio under NH_4_^+^ supply. (C) LR density in the seminal root. Seedlings were grown under NH_4_^+^ and partial nitrate nutrition (PNN) with or without application of SNP (2.5 μM, cv. Nanguang; 5 μM, cv. Eilo), cPTIO (80 μM for the two cultivars), or tungstate (Tu; 50 μM, cv. Nanguang; 100 μM, cv. Eilo) for 14 d in hydroponic media. Scale bar=1mm. Data are means ± standard error (SE), and bars with different letters indicate significant differences at *P*<0.05, as determined by ANOVA.

**Fig. 3.**
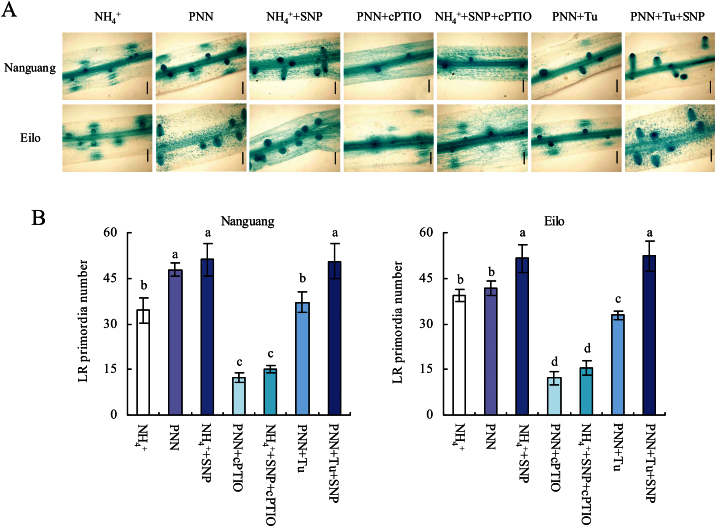
Lateral root (LR) primordia number in cvs Nanguang and Elio. Seedlings were grown under NH_4_^+^ and partial nitrate nutrition (PNN) with or without application of SNP (2.5 μM, cv. Nanguang; 5 μM, cv. Eilo), cPTIO (80 μM for the two cultivars), or tungstate (Tu; 50 μM, cv. Nanguang; 100 μM, cv. Eilo) for 14 d in hydroponic media. Scale bar=200 μm. Data are means ± standard error (SE), and bars with different letters indicate significant differences at *P*<0.05, as determined by ANOVA.

The effects of an NO donor and scavenger (SNP and cPTIO) on LR density and primordia were examined to determine whether the effect of PNN on the LR density in cv. Nanguang was mediated by NO. In cv. Nanguang, LR density increased upon application of 1–10 μM SNP. In cv. Elio, LR density increased slightly upon application of up to 5 μM SNP, and increased significantly with concentrations ≥5 μM (Supplementary Fig. S1 at *JXB* online). Thus, different SNP concentrations (2.5 μM, cv. Nanguang; 5 μM, cv. Elio) were used in subsequent analyses (Figs 2, 3). Application of SNP markedly induced NO accumulation and LR density and primordia in both rice cultivars (Fig. 3). Treatment with the NO scavenger cPTIO resulted in inhibition of PNN- and SNP-induced LR density and primordia in cv. Nanguang to a similar level. In cv. Elio, cPTIO application markedly decreased NO accumulation and LR density and primordia (Figs 2, 3). These results suggested that NO participated in LR formation in both rice cultivars and that NO participated in PNN-induced LR formation in cv. Nanguang but not in cv. Eilo.

### The PNN-induced NO accumulation is derived mainly from an NIA2-dependent NR source

**Fig. 4.**
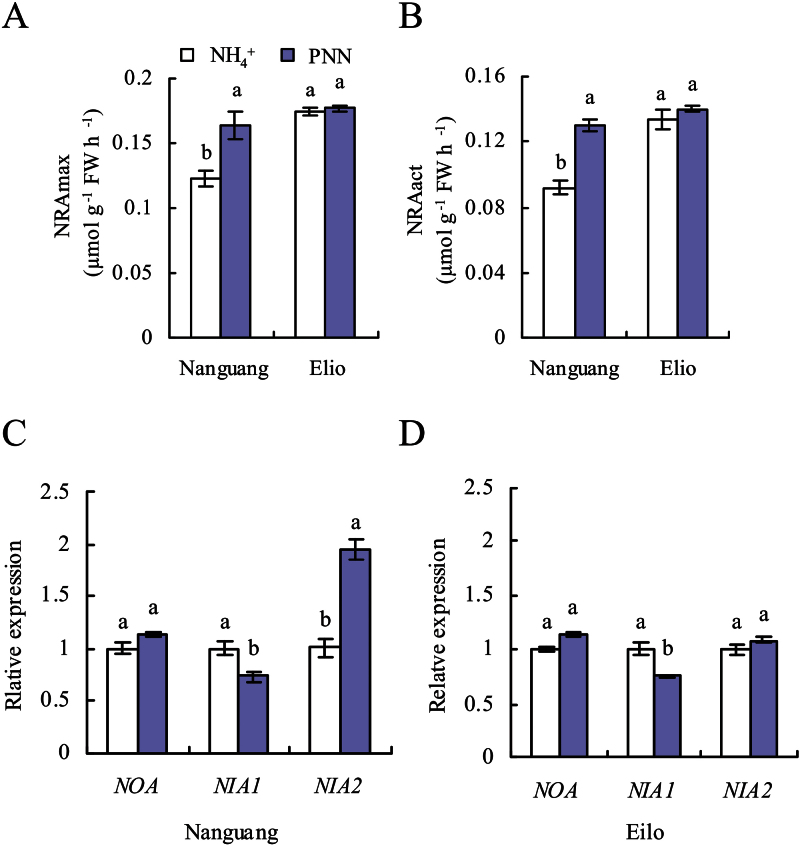
Maximum and active nitrate reductase activities (NRAmax and NRAact), and qRT–PCR analysis of *OsNOA*, *OsNIA1*, and *OsNIA2* expression in the roots of cvs Nanguang and Elio. (A and B) NRAmax and NRAact in cvs Nanguang and Elio. (C and D) *OsNOA*, *OsNIA1*, and *OsNIA2* expression levels in cvs Nanguang (C) and Elio (D). Seedlings were grown under NH_4_^+^ and partial nitrate nutrition (PNN) for 14 d (A and B) and 2 d (C and D) in hydroponic media. Relative mRNA levels were normalized to *OsACT*. Data are means ± standard error (SE), and bars with different letters in the four treatments (A and B) and the same genes (C and D) indicate significant differences at *P*<0.05, as determined by ANOVA.

NRAmax and NRAact levels in the roots of cv. Nanguang increased by 33% and 41% under PNN compared with NH_4_^+^ treatment, while no differences were observed in cv. Elio (Fig. 4A, B). Cao *et al.* (2008) reported that *NIA2* expression was highly induced by nitrate in rice cultivars. *NIA2* expression in the root of cv. Nanguang increased ~2-fold under PNN treatment compared with NH_4_^+^ treatment, consistent with the findings of Cao *et al.* (2008) (Fig. 4C). No difference in *NIA2* expression was observed in the roots of cv. Elio. However, *NIA1* expression decreased slightly in the roots of both rice cultivars (Fig. 4C, D). This change in *NIA2* expression is in agreement with that of NRAmax and NRAact, indicating that PNN affected NR at the transcriptional level. Upon treatment of cv. Nanguang with the NR inhibitor Tu under PNN conditions, the NO-associated green fluorescence and the LR density and primordia decreased to levels similar to those under NH_4_^+^ treatment (Figs 2, 3). Similarly, Tu application significantly decreased DAF fluorescence, and LR density and primordia in cv. Elio. Furthermore, *NOA* expression in the two rice cultivars did not differ between the two N treatments (Fig. 4C, D). These results suggested that *NIA2*-dependent NR-derived NO is a key signal in PNN-induced LR formation in cv. Nanguang.

### Role of NO in PNN-enhanced biomass and N accumulation

**Fig. 5.**
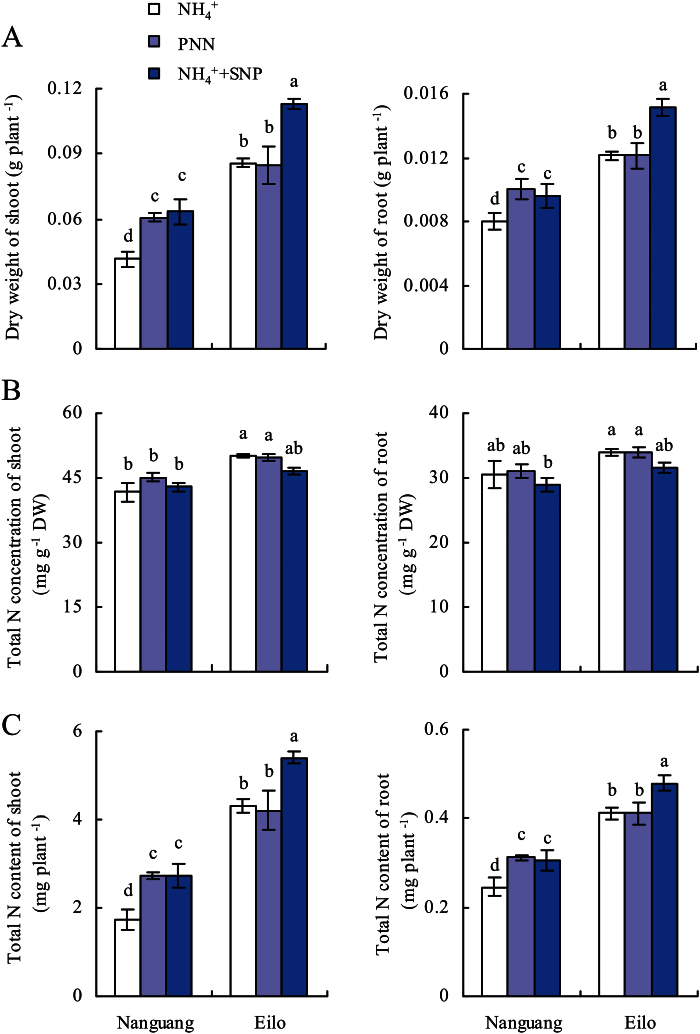
Growth and N accumulation of cvs Nanguang and Elio in response to partial nitrate nutrition (PNN) and SNP. Dry weight (A), total N concentration (B), and total N content (C) in the shoots and roots of cvs Nanguang and Elio. Rice seedlings were grown under NH_4_^+^ or PNN for 14 d in hydroponic media with or without SNP (2.5 μM, cv. Nanguang; 5 μM, cv. Eilo). Data are means ± standard error (SE), and bars with different letters indicate significant differences at *P*<0.05, as determined by ANOVA.

As reported previously (Duan *et al*., 2007*a*, b; Cao *et al.*, 2008; Song *et al.*, 2013*b*), PNN treatment increased the dry weight and N accumulation of cv. Nanguang, but not cv. Eilo. For example, the dry weights of shoots and roots increased by 46% and 25%, respectively, in cv. Nanguang with PNN treatment compared with NH_4_^+^ treatment (Fig. 5A, B). However, no differences were recorded for cv. Elio. Interestingly, SNP application in addition to NH_4_^+^ nutrition increased the dry weights of shoots and roots in rice cultivars. The total N concentration did not differ in each rice cultivar under the three treatments (Fig. 5B). However, the total N contents of shoots and roots increased under SNP application in both cvs Nanguang and Eilo. These results indicated that NO might be involved in N accumulation of cv. Nanguang under PNN treatment. For example, PNN treatment increased the total N contents of shoots and roots in cv. Nanguang by ~57% and 27%, respectively, and SNP application by ~57% and 25%, respectively (Fig. 5C). In addition, application of SNP induced the total N contents of shoots and roots in cv. Elio. These results supported the involvement of NO as a key signal in PNN-induced N accumulation in rice.

### Role of NO in PNN-enhanced N uptake rate

**Fig. 6.**
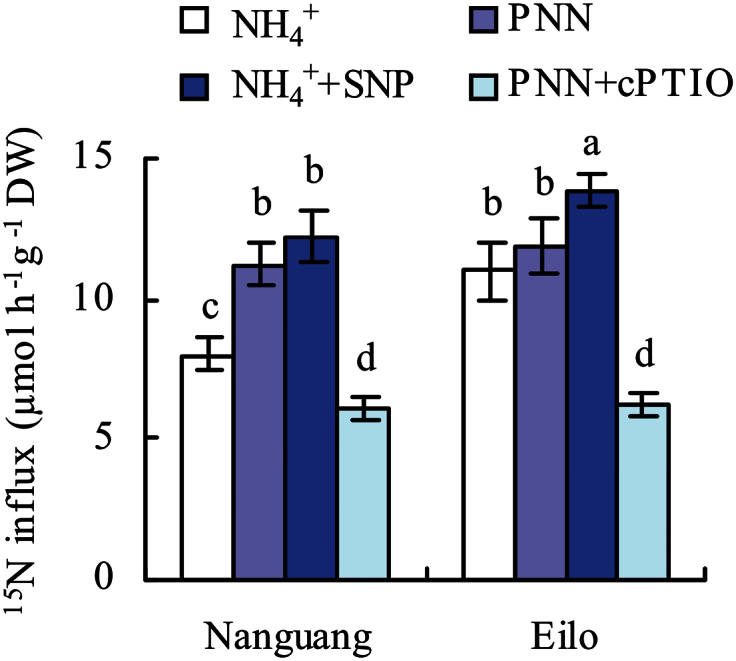
^15^N influx rates of the roots of cvs Nanguang and Eilo as measured by ^15^NH_4_^+^ and ^15^NO_3_^–^. Seedlings were grown under 1.43mM NH_4_^+^ for 14 d and then N starved for 3 d, before being resupplied with ^15^NH_4_^+^, ^15^NH_4_^+^/^15^NO_3_^–^ (75/25, PNN), ^15^NH_4_^+^+SNP (2.5 μM, cv. Nanguang; 5 μM, cv. Eilo), and ^15^NH_4_^+^/^15^NO_3_^–^ (75/25, PNN)+cPTIO (80 μM for the two cultivars) for 10min before harvest. Data are means ± standard error (SE), and bars with different letters indicate significant differences at *P*<0.05, as determined by ANOVA.

PNN treatment increased the rate of ^15^NH_4_^+^ uptake in cv. Nanguang, but not cv. Elio (Duan *et al.*, 2007*b*). To explore the effects of NO on NH_4_^+^ and NO_3_^–^ influx into intact plants, seedlings of both rice cultivars were incubated in solutions containing ^15^NH_4_^+^, ^15^NH_4_^+^/^15^NO_3_^–^ (PNN),^ 15^NH_4_^+^+SNP, and ^15^NH_4_^+^/^15^NO_3_^–^ (PNN)+cPTIO for 10min. Figure 6 shows that PNN treatment increased ^15^N influx in cv. Nanguang, but not cv. Eilo. Meanwhile, application of SNP in addition to NH_4_^+^ nutrition increased the ^15^N influx, and cTPIO application in addition to PNN reduced the ^15^N influx in both rice cultivars (Fig. 6). These results supported the involvement of NO as a key signal in PNN-induced N uptake rate in rice.

### NO donor and scavenger affected *OsAMT1–3*, *OsNRT2*, and *OsNAR2* gene expression

**Fig. 7.**
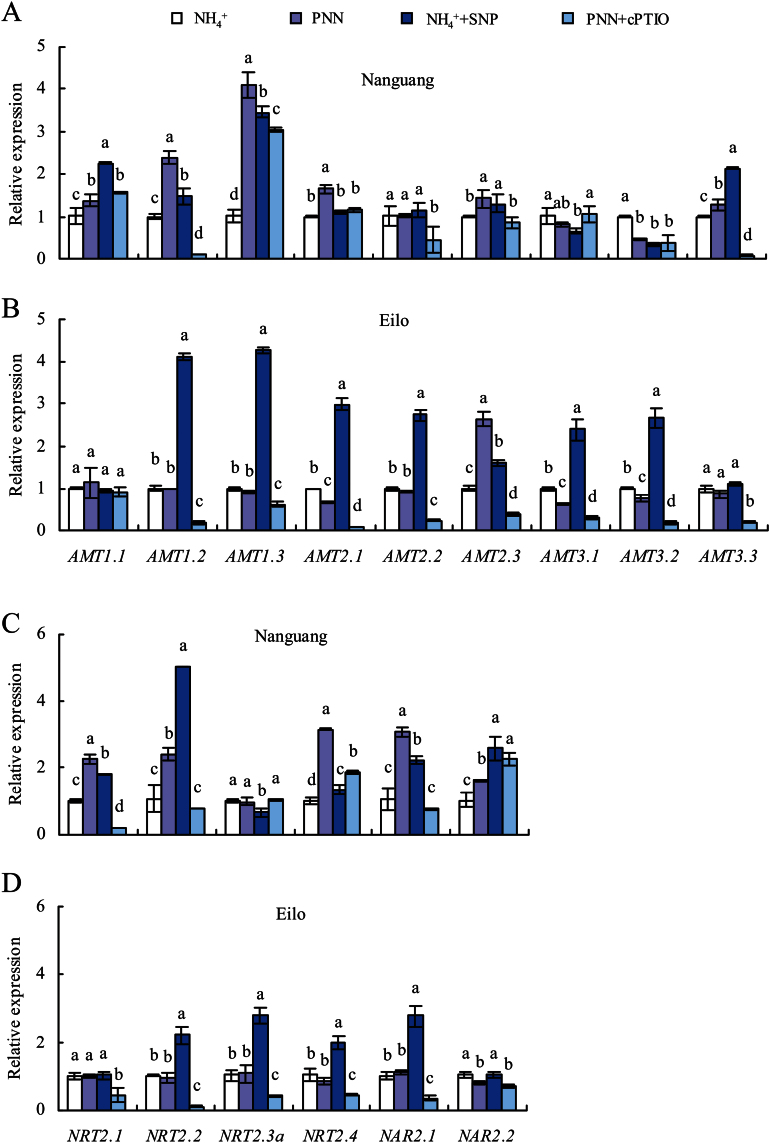
qRT–PCR analysis of *AMT1–3*, *NRT2*, and *NAR2* family genes in cvs Nanguang (A and C) and Elio (B and D) rice seedlings. Seedlings were grown in nutrient solution containing NH_4_^+^ only and partial nitrate nutrition (PNN) with or without SNP (2.5 μM, cv. Nanguang; 5 μM, cv. Eilo) and cPTIO (80 μM for tthe two cultivars) for 2 d in hydroponic media. Relative mRNA levels were normalized to *OsACT*. Data are means ± standard error (SE), and bars with different letters indicate significant differences at *P*<0.05 as determined by ANOVA.

The expression levels of *AMT*, *NRT2*, and *NAR2* genes were analysed using qRT–PCR (Fig. 7). The expression of *AMT1.1–3*, *AMT2.1*, *AMT2.3*, *AMT3.3*, *NRT2.1–2*, *NRT2.4*, and *NAR2.1–2* were significantly increased under PNN compared with NH_4_^+^ in cv. Nanguang (Fig. 7A, C). SNP application under NH_4_^+^ conditions induced transcript levels of most genes in cv. Nanguang. Conversely, the application of cPTIO under PNN conditions decreased the expression levels of most genes in cv. Nanguang, with the exception of *AMT3.1*, *NRT2.3a*, and *NAR2.2* (Fig. 7A, C). These results indicated that NO increased the N uptake rate under PNN treatment by up-regulating the expression of most nitrate and ammonium transporter genes.

Compared with cv. Nanguang, the two N treatments affected the expression levels of few genes in cv. Elio. Only the expression of *AMT2.3* was significantly increased by PNN in cv. Eilo compared with the NH_4_^+^ treatment (Fig. 7B). Application of SNP under NH_4_^+^ conditions increased the expression of *AMT1.2–3*, *AMT2.1–3*, *AMT3.1–2*, *NRT2.2–4*, and *NAR2.1* in cv. Eilo compared with NH_4_^+^ treatment alone (Fig. 7B, D). Meanwhile, the application of cPTIO decreased the expression levels of most genes in cv. Elio, with the exception of *AMT1.1*. These results showed that, for cv. Elio, PNN did not increase the N uptake rate and did up-regulate the expression level of *AMT2.3*. NO also increased the expression levels of most nitrate and ammonium transporters.

## Discussion

Nitrogen is a major limiting element of plant growth, and crops depend on intense fertilization, which has potential negative effects on the environment (Xu *et al.*, 2012). The situation is worse in China due to the added pressure of a large population (Zhang *et al.*, 2009). The identification of crop cultivars with improved nutrient acquisition efficiencies in low-input farming systems continues to be an important goal for plant scientists (Robertson and Vitousek, 2009; Xu *et al.*, 2012). Due to its lower 1000-grain weight, the rice cv. Nanguang, a high-NUE and high-nitrate-response cultivar, had less biomass accumulation than cv. Elio, a low-NUE and low-nitrate-response cultivar, during the early growth stage. However, a higher growth rate was recorded in cv. Nanguang, resulting in higher biomass accumulation and grain yield than in cv. Elio during the maturity stage (Duan *et al.*, 2007*b*; Zhang *et al.*, 2007; Zhang *et al.*, 2009). Previous studies have shown that PNN increased the N uptake efficiency, mainly by increasing root growth and the NH_4_^+^ uptake rate, which was attributed to high NUE in cv. Nanguang relative to sole NH_4_^+^ nutrition (Duan *et al*., 2007*a*, b; Li *et al.*, 2008; Zhang *et al.*, 2011; Song *et al.*, 2011*a*, b, 2013*a*). However, the mechanism(s) by which rice senses external nitrate conditions and the corresponding signal transduction pathway remains unclear.

Together with its role as an essential nutrient, nitrate acts as a signal to trigger a number of molecular and physiological events that lead to the overall response of a plant to N availability (Gojon *et al.*, 2011). More recently, molecular NO has been reported to be a key signal in nitrate sensing as an early response to nitrate supply in maize roots (Trevisan *et al.*, 2011; Manoli *et al.*, 2014). The nitrate supply caused consistent increases in DAF fluorescence during the first minutes. The role of *NOA* during NO synthesis remains controversial. It has been demonstrated that *Arabidopsis* *NOA1* does not possess NOS activity, being instead a GTPase (Moreau *et al.*, 2008). However, Schlicht *et al.* (2013) identified *noa1* mutants that contained lower NO levels in the root relative to the wild type. In this study, the *NOA* gene, a homologue of *NOA1* in *Arabidopsis*, showed a similar transcript level in the two rice cultivars between the two N treatments (Fig. 4C, D). It is possible that PNN did not increase NO levels through the NOS pathway; alternatively, *NOA* may not play a role in NO synthesis. Moreover, the application of chemicals that interfere with NO biosynthesis and scavenging provided further evidence that NO was produced by NR rather than by NOS.

Nitrate reductase is also involved in NO production in response to biotic and abiotic stresses in several upland plants (Modolo *et al.*, 2005; Srivastava *et al.*, 2009; Zhao *et al.*, 2009; Freschi *et al.*, 2010; Kolbert *et al.*, 2010). The observations that *NIA1* (but not *NIA2*) expression was sensitive to hormonal and developmental cues (Yu *et al.*, 1998), and the role of *NIA1* in NO production in guard cells (Bright *et al.*, 2006) and in response to cold acclimation (Zhao *et al.*, 2009), support that *NIA1* is a key component of NR-mediated NO production. It is known that nitrate rapidly induces the expression of NR genes in plants. However, the NR genes responsible for NR-mediated NO production in response to nitrate remain unclear.

In this study, it was shown that NO production was generated by NR in response to nitrate nutrition in rice roots. First, NRAmax and NRAact were induced significantly in response to nitrate supply in cv. Nanguang, but not cv. Elio, compared with in the presence of NH_4_^+^ nutrition. The NRAact to NRAmax ratio remained constant under the two N treatments, indicating that PNN did not regulate NR at the post-translational level. The significant increase in NO accumulation in the root tip and LR zone of rice plants was consistent with the pattern of NR activity. Secondly, for rice plants, the transcript level of *NIA2* was considerably higher than that of *NIA1* (Fan *et al.*, 2007; Cao *et al.*, 2008), consistent with the results in *Arabidopsis* (Wilkinson and Crawford, 1991). The expression of *NIA2* in cv. Nanguang was markedly induced by the nitrate supply and correlated well with tissue NR activity, consistent with the results of Cao *et al.* (2008). However, different responses of *NIA1* mRNA levels to PNN in rice plants were recorded in the present study and by Cao *et al.* (2008), possibly resulting from the fact that ammonium is the predominant N supply form for this species. Although a strong correlation between *NIA2* mRNA levels and NRA was observed, further experiments are required to evaluate the effects of PNN-induced changes. Thirdly, application of the NR inhibitor Tu in addition to PNN reduced DAF fluorescence in both cvs Nanguang and Eilo, suggestive of the involvement of NR in NO generation in rice roots.

The most important example of plant plasticity with regard to N availability is their ability to rearrange their root architecture and maximize nutrient capture (Lόpez-Bucio *et al.*, 2003; Zhang *et al.*, 2007; Forde, 2014). Nitrate affects root development by regulating the growth of LRs in a manner dependent on its localization and external concentration (Zhang and Forde, 1998; Zhang *et al.*, 1999; Linkohr *et al.*, 2002; Zhang *et al.*, 2007; Ruffel *et al.*, 2011; Mounier *et al.*, 2013; Forde, 2014). Due to the previous results showing PNN-induced LR density during a 14 d experimental stage with no change in seminal and adventitious roots relative to sole NH_4_^+^ nutrition, the effects of nitrate on NO generation in the root tip and LR zone were examined (Fig. 1C). These results showed marked and specific induction of LR initiation in seminal roots of rice seedlings supplied with NH_4_^+^+SNP relative to those supplied with NH_4_^+^ alone, and considerable inhibition upon the application of cPTIO or Tu with the PNN. This result is in agreement with the change in relative NO fluorescence in the two rice cultivars. These results suggest that the NO generated by NR contributes to LR initiation in response to PNN.

Nitrate acts both as a nutrient and as a signal that regulates plant N acquisition and metabolism (Crawford, 1995; Stitt, 1999; Forde, 2002). Early studies on nitrate signalling showed that nitrate induced expression of NR and nitrate transporters in the nitrate assimilation pathway (Zielke and Filner, 1971; Somers *et al.*, 1983; Stitt, 1999; Wang *et al.*, 2000). Studies on nitrate as a nutrient suggested that its ability to prevent acidification of the root medium plays an important role in maintaining the plasma membrane potential and therefore the physiological patterns of NH_4_^+^ uptake (Babourina *et al.*, 2007). Previous reports have shown that PNN induced increased N accumulation by enhancing the N-uptake efficiency in rice cultivars with high NUE (Duan *et al.*, 2007*b*). In agreement with this, N accumulation in cv. Nanguang increased by 53% by PNN or SNP in addition to NH_4_^+^ nutrition, but was enhanced by SNP treatment only in cv. Elio. These effects were probably due to NO induction of N uptake and assimilation. NR activity in cabbage roots was consistently higher following SNP treatment than in the control, while the NR protein content was unaffected by SNP (Du *et al.*, 2008), indicating that NO stimulated NR at the post-translational level. Similarly, in beech seedlings, the NO-mediated increase in the ammonium uptake rate was mediated at the level of protein modification rather than gene expression (Simon *et al.*, 2009). However, no such phenomenon was detected in Scots pine seedlings (Simon *et al.*, 2013), suggesting that the effects of NO on N uptake differ among plant species. The present short-duration ^15^N labelling experiments showed a significant induction of ^15^N influx in rice seedlings supplied with PNN (^15^NH_4_^+^/^15^NO_3_^–^) or ^15^NH_4_^+^+SNP relative to NH_4_^+^ nutrition alone, and a marked inhibition upon application of cPTIO in addition to the PNN supply. Interestingly, expression of high-affinity ammonium transporters (*AMT1.2–1.3*) and high-affinity nitrate transporters (*NRT2.2* and *NAR2.1*) in both rice cultivars was induced significantly by SNP, but reduced by cPTIO application. This result is in agreement with the changes in ^15^N influx, suggesting that NO regulated N uptake at the transcriptional level.

Taken together, these results suggest that NO generated by NR plays a pivotal role in improving N acquisition capacity by modulating LR initiation and the N-uptake rate. This may represent a strategy by which rice plants adapt to variations in nitrate supply and increase their NUE.

## Supplementary data

Supplementary data are available at *JXB* online.

Supplementary Figure S1. Effect of the NO donor SNP on LR density of seminal roots.

Supplementary Figure S2. Effect of the NR inhibitor tungstate on LR density of seminal roots.

Supplementary Table S1. The primers for qRT–PCR of *OsNOA*, *OsNIA1*, and *OsNIA2* genes.

Supplementary Table S2. The primers for qRT–PCR of *OsAMT1–3*, *OsNRT2*, and *OsNAR2* genes.

Supplementary Data
