# Supplementary material for: Nitric oxide generated by nitrate reductase increases nitrogen uptake capacity by inducing lateral root formation and inorganic nitrogen uptake under partial nitrate nutrition in rice
Source: J Exp Bot. 2015 Mar 17;66(9):2449–59. doi: 10.1093/jxb/erv030 (PMC4986861; doi:10.1093/jxb/erv030)
Supplement: Supplementary Data [file supp_66_9_2449__index.html]

Nitric oxide generated by nitrate reductase increases nitrogen uptake capacity by inducing lateral root formation and inorganic nitrogen uptake under partial nitrate nutrition in rice — Nitric oxide generated by nitrate reductase increases nitrogen uptake capacity by inducing lateral root formation and inorganic nitrogen uptake under partial nitrate nutrition in rice — Supplementary Data 

# Nitric oxide generated by nitrate reductase increases nitrogen uptake capacity by inducing lateral root formation and inorganic nitrogen uptake under partial nitrate nutrition in rice

## Supplementary Data

Data files

**Files in this Data Supplement:**

- Supplementary Data - Supplementary Data
