# Supplementary material for: Nitric oxide generated by nitrate reductase increases nitrogen uptake capacity by inducing lateral root formation and inorganic nitrogen uptake under partial nitrate nutrition in rice
Source: J Exp Bot. 2015 Mar 17;66(9):2449–59. doi: 10.1093/jxb/erv030 (PMC4986861; doi:10.1093/jxb/erv030)
Supplement: Supplementary Data [file supp_erv030_jexbot137000_file001.pdf]

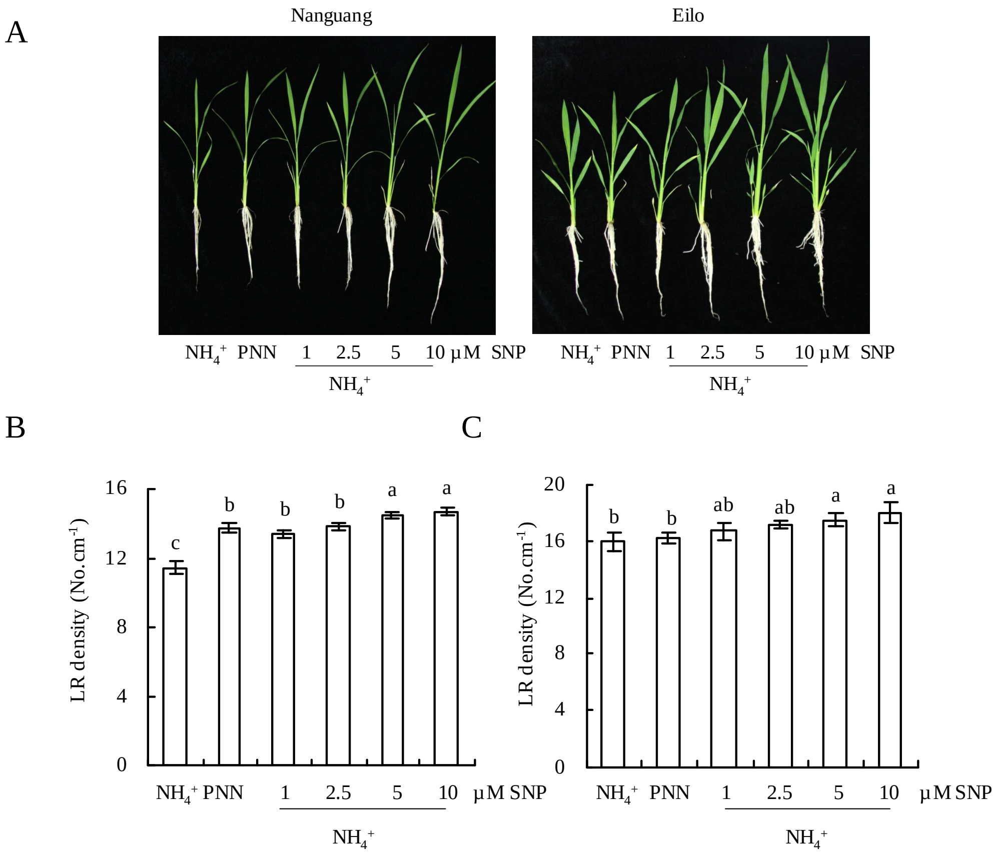

Fig. S1 Effect of NO donor SNP on LR density of seminal root in cvs. Nanguang (B) and Elio (C). (A) Appearence of seedlings treated with NH_4^+, partial nitrate nutrition (PNN) and NH_4^++SNP. Seedlings were grown in nutrient solution that contained NH_4^+-only and PNN with or without NO donor SNP for 14 days. Data are means ± SE and bars with different letters indicate significant differences at $P < 0.05$, as determined by ANOVA.

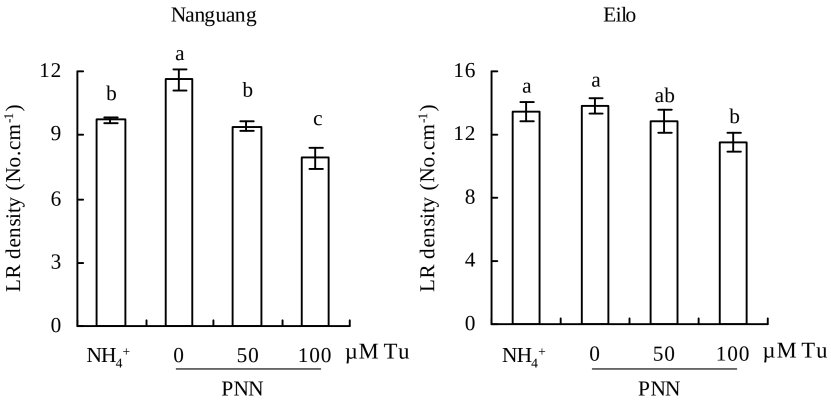

Fig. S2 Effect of NR inhibitor tungstate (Tu) on LR density of seminal root in cvs Nanguang and Elio. Seedlings were grown in nutrient solution that contained NH_4^+-only and PNN with NR inhibitor tungstate varying from 0 to 100μM for 14 days. Data are means ± SE and bars with different letters indicate significant differences at $P < 0.05$, as determined by ANOVA.

Table S1 The primers for qRT-PCR of *OsNOA*, *OsNIA1*, *OsNIA2* genes and *OsActin*.

Gene	Accession number	Primer sequence
OsNOA	Os02g0104700	5'-TGCTTCTGTGGTTGGGAC-3'
		5'-TCTAAGGGCACGGTGTTT-3'
OsNIA1	AK102178	5'-CCAATTCTTTCATCGTGTTCT-3'
		5'-CATGCAGCATTTCGTTTCT-3'
OsNIA2	AK102363	5'-ACTGGTGCTGGTGCTTCTGG-3'
		5'-CGGCTGGGTGTTGAGGGACT-3'
OsACT	LOC_Os03g50890	5'-CAACACCCCTGCTATGTACG-3'
		5'-CATCACCAGAGTCCAACACAA-3'

Table S2 The primers for qRT-PCR of *OsAMT1-3*, *OsNRT2* and *OsNAR2* genes.

Gene	Accession number	Primer sequence
OsAMT1.1	LOC_Os04g43070	5'-AGCGAAGGAAGAAATCACG-3'
		5'-CCAAACAGAAACTGGCAATC-3'
OsAMT1.2	LOC_Os02g40710	5'-TTCTACGTGCTGCACAGGTTC-3'
		5'-TTGCTCCGGCGACTTTCT-3'
OsAMT1.3	LOC_Os02g40730	5'-GTCTAGTGGAACCGGAGGAG-3'
		5'-CCTATTATACAATCACGAAACCTG-3'
OsAMT2.1	LOC_Os05g39240	5'-CTGGCTCCTCCTCTCCTACA-3'
		5'-CAGGATGTTGTTCGGTGAGA-3'
OsAMT2.2	LOC_Os01g61510	5'-GCCTCGACGTCATCTTCTTC-3'
		5'-TTGTGGAGGATCATCATGGA-3'
OsAMT2.3	LOC_Os01g61550	5'-GCCTCGACGTCATCTTCTTC-3'
		5'-GGAAGGTGGATTTCTTGTGC-3'
OsAMT3.1	LOC_Os01g65000	5'-CTCCCGCAGACGACGCAGTT-3'
		5'-GCCGACGGTGTAGGAGAAGGTG-3'
OsAMT3.2	LOC_Os03g62200	5'-CTCACCTTCTCCTACACCGTC-3'
		5'-ACCCCATCCATAGTAACCCTG-3'
OsAMT3.3	LOC_Os02g34580	5'-GCTGGCGCACTATTTGTCA-3'
		5'-CATTCTGTGTCACTCCTACA-3'
OsNRT2.1	LOC_Os02g02170	5'-CTTGTTGCAAACGGTGATGA-3'
		5'-GCCTCTCCCTTATTATACCTCCG-3'
OsNRT2.2	LOC_Os02g02190	5'-CGGAGCACGCCTAATTAAGAG-3'
		5'-CTCCATGACGACATACTCTAGATA-3'
OsNRT2.3a	LOC_Os01g50820	5'-CGCTGCTGCCGCTCATCCG-3'
		5'-CCGTGCCCATGGCCAGAC-3'
OsNRT2.4	LOC_Os01g36720	5'-AAAGGTCGCTGGGCGTGGTG-3'
		5'-CCTGGACCCGCTGAAGAAGAG-3'
OsNAR2.1	LOC_Os02g38230	5'-CAAGGACAAGGCGTGCCAG-3'
		5'-GCGATGGAGAAGGTGGAG-3'
OsNAR2.2	LOC_Os04g40410	5'-GACGACCTGAGCAAGGACAAG-3'
		5'-TGCCACCGTGTACTCGAACTT-3'
